# The Automated Bone Scan Index as a Predictor of Response to Prostate Radiotherapy in Men with Newly Diagnosed Metastatic Prostate Cancer: An Exploratory Analysis of STAMPEDE’s “M1|RT Comparison”

**DOI:** 10.1016/j.euo.2020.05.003

**Published:** 2020-08

**Authors:** Adnan Ali, Alex P. Hoyle, Christopher C. Parker, Christopher D. Brawley, Adrian Cook, Claire Amos, Joanna Calvert, Hassan Douis, Malcolm D. Mason, Gerhardt Attard, Mahesh K.B. Parmar, Matthew R. Sydes, Nicholas D. James, Noel W. Clarke, on behalf of the STAMPEDE investigators

**Affiliations:** a Genito-Urinary Cancer Research Group, Division of Cancer Sciences, The University of Manchester, Manchester, UK; b FASTMAN Centre of Prostate Cancer Excellence, Manchester Cancer Research Centre, Manchester, UK; c Department of Surgery, The Christie NHS Foundation Trust, Manchester, UK; d Department of Urology, The Salford NHS Foundation Trust, Manchester, UK; e Royal Marsden Hospital and The Institute of Cancer Research, London, UK; f MRC Clinical Trials Unit at UCL, Institute of Clinical Trials and Methodology, UCL, London, UK; g Department of Radiology, University Hospitals Birmingham NHS Foundation Trust, Birmingham, UK; h Cardiff University, Cardiff, UK; i UCL Cancer Institute, London, UK

**Keywords:** Metastatic, Hormone naïve, Radiotherapy, Imaging

## Abstract

**Background:**

Prostate radiotherapy (RT) is a first-line option for newly diagnosed men with low-burden metastatic prostate cancer. The current criterion to define this clinical state is based on manual bone metastasis counts, but enumeration of bone metastases is limited by interobserver variations, and it does not account for metastasis volume or lesional coalescence. The automated bone scan index (aBSI) is a quantitative method of evaluating bone metastatic burden in a standardised and reproducible manner.

**Objective:**

To evaluate whether aBSI has utility as a predictive imaging biomarker to define a newly diagnosed metastatic prostate cancer population that might benefit from the addition of prostate RT to standard of care (SOC) systemic therapy.

**Design, setting, and participants:**

This is an exploratory analysis of men with newly diagnosed metastatic prostate cancer randomised in a 1:1 ratio to either SOC or SOC + prostate RT within the STAMPEDE “M1|RT comparison”.

**Intervention:**

The SOC was lifelong androgen deprivation therapy, with up-front docetaxel permitted from December 2015. Men allocated RT received either a daily or a weekly schedule that was nominated before randomisation.

**Outcome measurements and statistical analysis:**

Baseline bone scans were evaluated retrospectively to calculate aBSI. We used overall (OS) and failure-free (FFS) survival as the end points. Treatment-aBSI interaction was evaluated using the multivariable fractional polynomial interaction (MFPI) and subpopulation treatment effect pattern plot. Further analysis was done in aBSI quartiles using Cox regression models adjusted for stratification factors.

**Results and limitations:**

: Baseline bone scans for 660 (SOC: 323 and SOC + RT: 337) of 2061 men randomised within the “M1|RT comparison” met the software requirements for aBSI calculation. The median age was 68 yr, median PSA was 100 ng/mL, median aBSI was 0.9, and median follow-up was 39 mo. Baseline patient characteristics including aBSI were balanced between the treatment groups. Using the MFPI procedure, there was evidence of aBSI-treatment interaction for OS (*p* = 0.04, MFPI procedure) and FFS (*p* <  0.01, MFPI procedure). Graphical evaluation of estimated treatment effect plots showed that the OS and FFS benefit from prostate RT was greatest in patients with a low aBSI. Further analysis in quartiles based on aBSI supported this finding.

**Conclusions:**

A low automated bone scan index is predictive of survival benefit associated with prostate RT in men with newly diagnosed metastatic prostate cancer.

**Patient summary:**

The widely used bone scan can be evaluated using an automated technique to potentially select men with newly diagnosed metastatic prostate cancer who might benefit from prostate radiotherapy.

## Take Home Message

In this exploratory analysis of 660 men with newly diagnosed metastatic prostate cancer randomised within a clinical trial, a low automated bone scan index was found to be predictive of overall and failure-free survival benefit from the addition of prostate radiotherapy to standard of care systemic therapy. This was also observed with secondary clinical end points.

## 1 Introduction

Bone is the commonest site of metastatic spread in prostate cancer [1], and traditionally, bone metastases have been evaluated conventionally using ^99m^Tc methylene diphosphonate (^99m^Tc-MDP) bone scintigraphs to assess the presence and extent of metastases. The extent or burden of skeletal involvement based on ^99m^Tc-MDP bone scans has been reported to be prognostic in prostate cancer [2]. Recently, the individual and combined analyses of two phase III randomised controlled trials have reported that the extent of bone metastases is predictive for selecting newly diagnosed metastatic prostate cancer patients and men with lower metastatic burden benefit from prostate radiotherapy (RT) [3], [4], [5]. Based on the results of these two trials, prostate RT is now considered an option for patients with low metastatic burden [6], [7], [8].

Counting bone metastases to ascertain metastatic burden is limited by interobserver variation and it is not quantitative, failing to account for lesional volume or coalescence [9], [10]. Therefore, a standardised quantitative imaging biomarker would be a better method for the quantification of metastatic disease burden in bone. Previous reports have shown the artificial neural network–based automated bone scan index (aBSI) to be accurate and reproducible in this regard [11], [12], providing a quantitative assessment of bone metastasis burden as a fraction of skeletal weight. In prospective evaluation in a phase 3 clinical trial, it was an independent prognostic factor in metastatic castration-resistant prostate cancer [13]. Herein, we sought to determine its utility as a predictive imaging biomarker for defining a population of men newly diagnosed with metastatic prostate cancer who might benefit from the addition of prostate RT to standard of care (SOC) systemic therapies.

## 2 Patients and methods

### 2.1 Trial design and conduct

STAMPEDE is a multiarm, multistage trial that enrols men with advanced high-risk or metastatic prostate cancer. The trial is registered as NCT 00268476 and ISRCTN 78818544, and has the relevant regulatory, national ethics, and local practical site approval. All patients provided written informed consent.

Herein, we focus on patients who were randomised within STAMPEDE “M1|RT comparison”, that is, SOC (arm A) or SOC and prostate RT (arm H) [4]. Briefly, patients with newly diagnosed metastatic prostate cancer, with no previous radical treatment and no contraindication to RT, were eligible for this comparison. There were no age restrictions; patients had to be fit for chemotherapy and have no significant cardiovascular history. Patients underwent baseline imaging prior to randomisation as per study protocol, which included computed tomography or magnetic resonance imaging (MRI) of the pelvis and abdomen; bone scan or equivalent, for example, whole-body MRI; and chest x-ray if chest was not included in the computed tomography or MRI. Randomisation was stratified according to centre, age at randomisation (<70 vs ≥70 yr), World Health Organization (WHO) performance status (0 vs 1 or 2), nodal involvement (N0 vs N1 vs NX), type of androgen deprivation therapy (ADT), and use of aspirin or nonsteroidal anti-inflammatory drugs. Planned docetaxel use was added as a stratification factor on 17 December 2015. Randomisation was in a 1:1 ratio to receive SOC systemic therapy alone or SOC plus prostate RT. The SOC was lifelong ADT, with up-front docetaxel permitted from 17 December 2015. Docetaxel was given as six 3-weekly cycles of 75 mg/m², with or without prednisolone 10 mg daily. Men allocated RT received either a daily (55 Gy in 20 fractions over 4 wk) or a weekly (36 Gy in six fractions over 6 wk) schedule that was nominated before randomisation. Full details are provided in the study protocol, which can be found at http://www.stampedetrial.org.

### 2.2 Image analysis

The aBSI was calculated blinded to treatment assignment and outcome data using the Exini aBSI v3.2.1 software (EXINI Diagnostics, Lund, Sweden). Anterior and posterior Digital Imaging and Communications in Medicine (DICOM) images of baseline planar whole-body bone scans, which met the image and the pixel compression requirements (Supplementary methods), were evaluated retrospectively to calculate aBSI as described previously [13], [14]. Briefly, the skeleton is segmented into 12 regions; hotspots are detected and classified as metastatic or benign by an artificial neural network. Each metastatic hotspot size is divided by the size of the corresponding skeletal region and multiplied by a weight fraction constant. The aBSI is then calculated as the sum of all such hotspots. No manual correction to hotspot classification was applied unless it represented a large urinary bladder, a urinary catheter, or tracer contamination.

### 2.3 Outcomes

We used the trial’s primary and intermediate outcome measures—overall (OS) and failure-free (FFS) survival, respectively. OS was defined as the time from randomisation to death from any cause and FFS as the time from randomisation to the first of the following events: biochemical failure, progression either locally in lymph nodes or in distant metastases, or death from prostate cancer [4]. Biochemical failure was defined as a rise in PSA above its lowest reported level within 24 wk after enrolment of 50% and to at least 4 ng/mL; patients without a fall of 50% were considered to have biochemical failure at time zero. Secondary outcome measures, progression-free survival (defined as FFS but without biochemical events), metastatic progression-free survival (defined as the time from randomisation to new metastases or progression of existing metastases or death), prostate cancer–specific survival, and symptomatic local event–free survival (defined as any of the following: urinary tract infection, new urinary catheterisation, acute kidney injury, transurethral resection of the prostate, urinary tract obstruction, ureteric stent, nephrostomy, colostomy, and surgery for bowel obstruction) were also evaluated. Patients without the event of interest were censored at the time they were last known to be event free. The outcome dataset, frozen for the published STAMPEDE “M1|RT comparison”, was used for survival analyses [4].

### 2.4 Statistical analyses

To evaluate whether the effect of treatment varied by aBSI (treatment-aBSI interaction), a multivariable fractional polynomial interaction (MFPI) test using a nested Cox regression model was performed. This was to improve the statistical power for detecting interaction of a continuous variable with treatment [15] and to avoid arbitrary categorisation [16]. For the MFPI analyses, aBSI was modelled as a continuous variable using a second-degree fractional polynomial functional form separately for each treatment group using the same powers. The *p* value from a likelihood ratio test of treatment-aBSI interaction is presented. The MFPI model-estimated treatment effect as a function of aBSI was plotted graphically as hazard ratio (HR) and 95% confidence interval (CI). Further technical details regarding the MFPI have been published previously [17], [18].

The effect of treatment relative to aBSI was also assessed graphically using the subpopulation treatment effect pattern plot (STEPP) [19]. The tail-oriented version of STEPP was used, in which 2*g* – 1 overlapping subpopulations are created based on a parameter *g*. Within each subpopulation, relative treatment effects were evaluated using Cox regression. Further details on how subpopulations are created have been described previously [19]. Graphical assessment was based on the evaluation of estimated treatment effect in relation to aBSI: aBSI-treatment interaction would be manifested as a nonhorizontal line, that is, estimated treatment effect varying by aBSI, whereas a line parallel to the *X*-axis would indicate a constant treatment effect across the range of aBSI values.

We conducted further analysis by dividing the cohort into four quartiles (Q1–Q4) based on aBSI, with Q1 being the lowest and Q4 being the highest aBSI quartile. Within each subgroup defined by an aBSI quartile, treatment effects were evaluated using Kaplan-Meier (KM) plots and estimated using adjusted Cox regression. Cox models were adjusted for minimisation factors used at randomisation: age (<70 or ≥70 yr), N stage (N0, N+, or NX), WHO performance status (0 or 1–2), use of nonsteroidal anti-inflammatory drugs or aspirin (uses either or none of these), planned use of docetaxel (yes or no); except for hospital and planned ADT. An HR of <1 favours prostate RT + SOC. Median follow-up was calculated by reverse censoring on death. All statistical analyses were performed using Stata v15.1 (StataCorp, College Station, TX, USA).

## 3 Results

**Fig. 1:**
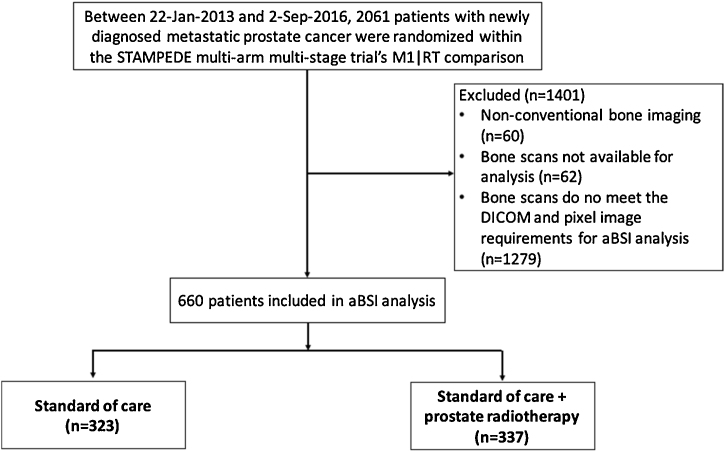
Flow diagram showing selection of patients. aBSI = automated bone scan index; DICOM = Digital Imaging and Communications in Medicine; RT = radiotherapy.

Baseline bone scans from 660 of the 2061 men with newly diagnosed metastatic prostate cancer, randomised between 22 January 2013 and 2 September 2016 within the STAMPEDE “M1|RT comparison”, had digital scan-based information usable for aBSI calculation and were included in this study (Fig. 1). Baseline characteristics of these patients were balanced between SOC and SOC + RT groups (Table 1) and were broadly similar to the M1|RT comparison (Supplementary Table 1). Median age at randomisation was 68 yr, and median PSA before ADT was 100 ng/mL. The median aBSI was 0.9 (interquartile range [IQR] 0.1–4.1) and was balanced between the treatment groups (*p* =  0.59, Wilcoxon rank-sum test). Median follow-up of the study cohort was 39 mo (IQR 24–48).

**Table 1:** Baseline characteristics of patients included in the aBSI cohort.

	SOC (*n* = 323)		SOC + RT (*n* = 337)	
	*n*	%	*n*	%
Age at randomisation				
Median	68		68	
IQR	63–73		63–73	
PSA (ng/mL) before ADT				
Median	94		111	
IQR	33–355		36–331	
WHO performance status				
0	248	77	252	75
1–2	75	23	85	25
Primary tumour stage				
≤T2	35	11	32	10
T3	195	60	205	61
T4	72	22	83	25
TX	21	7	17	5
Gleason score				
≤7	50	15	64	19
8–10	260	81	261	78
Unknown	13	4	12	4
Regional node status				
N0	93	29	111	33
N1	207	64	211	63
NX	23	7	15	4
Nominated RT schedule				
36 Gy in 6 f over 6 wk	158	49	179	53
55 Gy in 20 f over 4 wk	165	51	158	47
Planned docetaxel				
No	261	81	276	82
Yes	62	19	61	18
Metastatic sites				
Bone	289	89	306	91
NRLN	94	29	95	28
Lung	10	3	9	3
Liver	5	2	3	1
Other	11	3	8	2
Metastatic burden^a^				
Low	135	42	149	44
High	188	58	188	56
Number of bone metastases				
≤3	140	43	145	43
4–6	49	15	52	15
≥7	134	42	140	42
aBSI				
Median	1		0.8	
IQR	0.2–4.2			0.1–3.8

aBSI = automated bone scan index; ADT = androgen deprivation therapy; f = fractions; IQR = interquartile range; NRLN = nonregional lymph node; PSA = prostate-specific antigen; RT = radiotherapy; SOC = standard of care; WHO = World Health Organization.

a CHAARTED definition.

Using the MFPI procedure, there was evidence of aBSI-treatment interaction for OS (*p* =  0.04, MFPI procedure). Graphical assessment of estimated treatment effect against aBSI suggests that only patients with a low aBSI receive survival benefit associated with prostate RT (HR and 95% CI below 1; Supplementary Fig. 1A). Examination of estimated treatment effect in STEPP plots also showed a sloping nonhorizontal line for treatment effect, demonstrating that the treatment effect varies with aBSI and approximately reflects the estimated pattern of interaction (Supplementary Fig. 2A). Similarly, for FFS we found good evidence of aBSI-treatment interaction (*p* <  0.01, MFPI procedure). A plot of estimated treatment effect from MFPI analysis indicates that FFS benefit was greatest for patients with a low aBSI, with the upper 95% CI crossing the line of equivalence (HR: 1) just below 1 aBSI (Supplementary Fig. 1B). A similar pattern of changing treatment effect can be seen in the corresponding STEPP graph (Supplementary Fig. 2B).

**Fig. 2:**
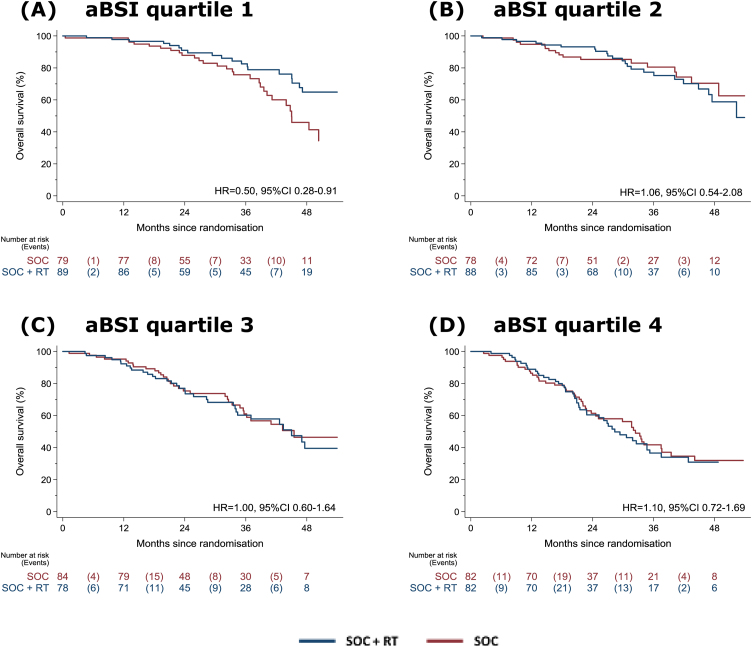
Kaplan-Meier plots for overall survival by treatment in (A–D) aBSI quartiles 1–4. aBSI = automated bone scan index; RT = radiotherapy; SOC = standard of care.

Further analysis was conducted in quartiles based on aBSI value, and aBSI was found to be balanced between the treatment groups within each aBSI quartile (Supplementary Tables 2–6). In quartile 1 comprising patients with aBSI <0.2, there was good evidence of survival benefit associated with the addition of prostate RT to SOC over SOC alone (HR = 0.50, 95%CI 0.28–0.91; 3-yr KM-estimated survival 76% with SOC vs 83% with SOC + RT; Table 2 and Fig. 2A). No evidence of survival benefit was noted with prostate RT in the other aBSI quartiles (HRs of 1.06 [95% CI 0.54–2.08], 1.00 [95% CI 0.60–1.64], and 1.10 [95% CI 0.72–1.69] in quartiles 2, 3, and 4 respectively; Table 2 and Fig. 2B–D).

**Table 2:** Summary of relative treatment effects in aBSI quartiles for overall and failure-free survival.

	No. of events/no. of patients		Hazard ratio (95% CI)^a^
	SOC	SOC + RT	
Overall survival			
aBSI quartile 1	29/79	19/89	0.50 (0.28–0.91)
aBSI quartile 2	17/78	23/88	1.06 (0.54–2.08)
aBSI quartile 3	32/84	32/78	1.00 (0.60–1.64)
aBSI quartile 4	45/82	46/82	1.10 (0.72–1.69)
Failure-free survival			
aBSI quartile 1	54/79	36/89	0.35 (0.22–0.54)
aBSI quartile 2	55/78	50/88	0.67 (0.44–1.00)
aBSI quartile 3	66/84	60/78	0.81 (0.56–1.16)
aBSI quartile 4	70/82	73/82	1.08 (0.76–1.52)

aBSI = automated bone scan index; CI = confidence interval; NSAID = nonsteroidal anti-inflammatory drug; RT = radiotherapy; SOC = standard of care; WHO PS = World Health Organization performance status.

a Adjusted for age (<70 or ≥70 yr), N stage (N0, N+, or NX), WHO PS (0 or 1–2), NSAID or aspirin use (uses either or none of these), and planned docetaxel use (yes or no).

**Fig. 3:**
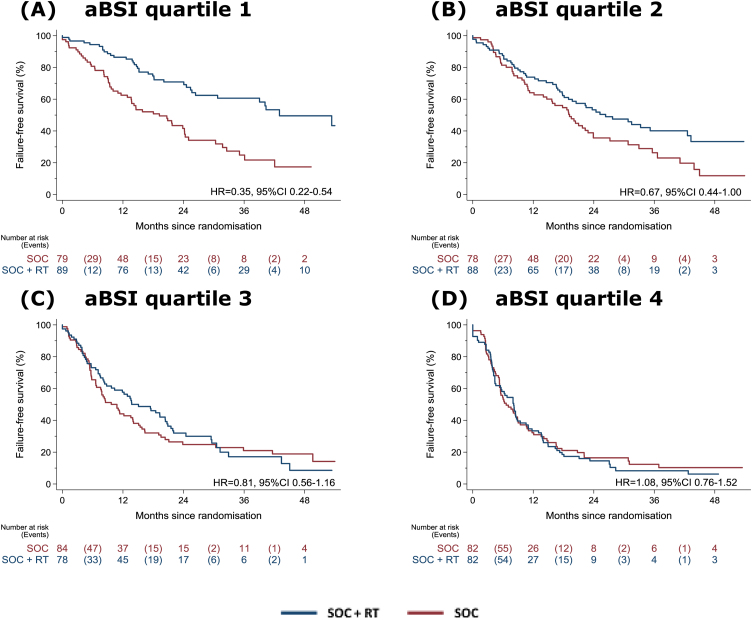
Kaplan-Meier plots for failure-free survival by treatment in (A–D) aBSI quartiles 1–4. aBSI = automated bone scan index; RT = radiotherapy; SOC = standard of care.

For FFS, we observed strong evidence of improved FFS with the use of prostate RT in quartile 1 with the lowest aBSI range. This becomes weaker in quartiles 2 and 3, and disappears in quartile 4 with the highest aBSI values. The estimated HR of adding prostate RT to SOC on FFS was 0.35 (95% CI 0.22–0.54; 3-yr KM-estimated FFS 26% with SOC vs 61% with SOC + RT; Table 2 and Fig. 3A). In quartiles 2, 3, and 4, the estimated HRs for the effect of prostate RT in comparison with SOC on FFS are 0.67 (95% CI 0.44–1.00), 0.81 (95% CI 0.56–1.16), and 1.08 (95% CI 0.76–1.52), respectively (Table 2 and Fig. 3B–D).

We evaluated the impact of prostate RT on secondary efficacy measures within aBSI quartiles. In aBSI quartile 1, there was evidence of benefit from adding prostate RT to progression-free survival (HR = 0.43, 95% CI 0.26–0.71), metastatic progression-free survival (HR = 0.50, 95% CI 0.30–0.85), prostate cancer–specific survival (sub-distribution HR = 0.37, 95% CI 0.17–0.81), and symptomatic local event-free survival (HR = 0.53, 95% CI 0.31–0.90). There was no evidence of benefit from the addition of prostate RT in aBSI quartiles 2–4 on any secondary outcome measures (Supplementary Table 7).

## 4 Discussion

In this study, we found that a low baseline aBSI in patients with newly diagnosed metastatic prostate cancer is *predictive* of survival benefit when prostate RT is added to SOC systemic therapy. The clinical implication of this study is that the widely available conventional bone scan can be evaluated using an observer-independent automated technique to select patients who would benefit from prostate RT as part of multimodality treatment.

Two trials have previously reported a beneficial effect of prostate RT in patients with low metastatic burden based on manual bone metastasis counts. The HORRAD trial that enrolled 432 patients showed, in a subgroup of 160 patients with fewer than five bone metastases, some evidence of OS benefit of the combination of prostate RT and ADT compared with ADT alone (HR = 0.68, 95% CI 0.42–1.10) [3]. However, this subgroup analysis was underpowered and the categorisation was done in groups based on bone metastases count as 1–4, 5–15, and >15. In the STAMPEDE “M1|RT” comparison, a prespecified, well-powered, and directionally hypothesised subgroup analysis based on the CHAARTED definition showed significant improvement in survival associated with the addition of prostate RT to SOC in patients with low metastatic burden (HR = 0.68, 95% CI 0.52–0.90) [4]. Further exploratory analysis of 1939 patients within the STAMPEDE M1|RT comparison has shown that manual bone metastasis counts are *predictive* of OS and FFS benefit, and this benefit is limited to patients with only non-regional lymph node metastasis (M1a) or three or fewer bone metastases regardless of axial or appendicular location without any visceral or other metastasis [20]. Given that aBSI is highly correlated with manual bone counts, the current findings based on aBSI are supported by previous large-scale studies based on manual counting.

This study also highlights that beyond counting the number of bone metastases, metastatic disease volume is another characteristic with potential to be clinically useful in selecting M1 patients for prostate RT. In this study, aBSI quartile 1 (aBSI <0.2) consisted predominantly of patients with three or fewer bone metastases (93%). However, these represented 55% of the men with three or fewer bone metastases; the other 45% belonged to aBSI quartiles 2 (37%) and 3 (8%) with higher aBSI. This suggests that although certain patients might meet the enumeration criteria, they might not meet the “aBSI volume” threshold. Most patients in this study had bone metastases, but a proportion also had bone, node, and visceral metastases alone or in combination. Quantification of nonosseous metastases is not addressed using the aBSI methodology, and the implications of this need to be studied further. Previous exploratory analysis of the STAMPEDE “M1|RT comparison” has demonstrated that patients with a negative bone scan and only nonregional lymph node metastases (M1a) benefit from prostate RT, while patients with any visceral metastasis (M1c) do not [20]. An exploratory analysis that accounts for site and volume of lymph node, bone, and visceral metastases is currently on-going within STAMPEDE. In parallel, imaging methodology in prostate cancer is developing rapidly, with newer techniques, such as prostate-specific membrane antigen positron emission tomography, whole-body MRI, and others, gaining popularity. Given the higher accuracy of novel imaging than conventional imaging, we would expect newer methodologies to detect more metastases [21]. However, the true relevance of these findings based on newer imaging in relation to outcomes from treatment is unknown. These newer imaging methods will need further detailed study to ascertain their true individual clinical relevance. Future studies can evaluate the prognostic and predictive relevance of quantitative imaging biomarkers.

Our study has limitations given its exploratory and retrospective nature. However, even after the exclusion of a number of patients whose bone scans could not be evaluated for aBSI because of a lack of uncompressed DICOM pixel data or only spot images, 660 fully categorised patients were available for the study. Baseline characteristics and follow-up survival data of the aBSI cohort and total trial population were determined to be similar. The use of the MFPI procedure allowed the use of all the available information from the continuous aBSI variable, and the results did not depend on any cut-point for an interaction test. Previous simulation studies have shown that the power of the MFPI procedure to identify an interaction is greater than the power of the common approach based on dichotomisation using cut-points [22], [23].

The aBSI method to evaluate bone metastatic burden has several advantages that are potentially useful for the patient and clinician. It has previously undergone analytical validation demonstrating rapid (<10 s per scan), accurate, and reproducible assessments of metastatic burden, minimising interobserver variability [11], [12]. Given the widespread availability of conventional bone scan and its use in randomised controlled trials, the quantitative aBSI method can potentially be used prospectively for stratifying patients in clinical trials evaluating local treatment for newly diagnosed metastatic prostate cancer. It can also be used to monitor treatment responses [24]. This can be done easily using technology that is available widely and by prospective implementation of standardised operating procedures to acquire, store, and analyse bone scan DICOM images by clinical teams undertaking the patient evaluation and treatment. A number of on-going trials are evaluating local and metastasis-directed treatments in metastatic prostate cancer [25]. These trials can further evaluate aBSI as a standardised method of metastatic burden quantification.

## 5 Conclusions

A low aBSI is predictive of survival benefit associated with the addition of prostate RT to SOC systemic therapies. This methodology has significant potential for use in the selection of newly diagnosed metastatic prostate cancer patients who might benefit from RT to the primary lesion.

  ***Author contributions*:** Noel W. Clarke had full access to all the data in the study and takes responsibility for the integrity of the data and the accuracy of the data analysis.

*Study concept and design*: Ali, James, Sydes, Clarke.

Acquisition of data: All authors.

Analysis and interpretation of data: All authors.

*Drafting of the manuscript*: Ali, Hoyle, Brawley, Sydes, Parmar, Parker, James, Clarke.

Critical revision of the manuscript for important intellectual content: All authors.

*Statistical analysis or provision of outcome data*: Ali, Brawley, Sydes, Clarke.

*Obtaining funding*: James, Parmar, Sydes, Clarke.

Administrative, technical, or material support: Amos, Calvert.

*Supervision*: James, Parmar, Sydes, Clarke.

*Other*: None.

  ***Financial disclosures:*** Noel W. Clarke certifies that all conflicts of interest, including specific financial interests and relationships and affiliations relevant to the subject matter or materials discussed in the manuscript (eg, employment/affiliation, grants or funding, consultancies, honoraria, stock ownership or options, expert testimony, royalties, or patents filed, received, or pending), are the following: None.

  ***Funding/Support and role of the sponsor*:** Research support for protocol was received from Cancer Research UK (CRUK_A12459), Medical Research Council (MRC_MC_UU_12023/25), Astellas, Clovis Oncology, Janssen, Novartis, Pfizer, and Sanofi-Aventis. Professor Christopher C. Parker and Professor Nicholas D. James acknowledge that this paper represents independent research part funded by the National Institute for Health Research (NIHR) Biomedical Research Centre at the Royal Marsden NHS Foundation Trust and the Institute of Cancer Research. The views expressed are those of the author(s) and not necessarily those of the NHS, the NIHR, or the Department of Health.

  ***Acknowledgements*:** We thank all the patients who have participated in the trial, everyone supporting them and the participating site and CTU staff, without whom this trial would not have been possible. A full list of acknowledgements is available in the STAMPEDE M1|RT comparison publication [4]. The project could not have been undertaken without centralised staging radiological investigations, which was undertaken at the Christie NHS Foundation Trust by the PACS team that helped establish the imaging centralisation platform.
