# Supplementary material for: The Automated Bone Scan Index as a Predictor of Response to Prostate Radiotherapy in Men with Newly Diagnosed Metastatic Prostate Cancer: An Exploratory Analysis of STAMPEDE’s “M1|RT Comparison”
Source: Eur Urol Oncol. 2020 Aug;3(4):412–9. doi: 10.1016/j.euo.2020.05.003 (PMC7443695; doi:10.1016/j.euo.2020.05.003)
Supplement: Supplementary file 1 [file mmc1.docx]

**Supplementary material**

1. **Supplementary methods:** Image requirements for aBSI analysis
2. **Supplementary Figure 1:** Treatment effect plots for automated bone scan index (aBSI) from a multivariable fractional polynomial interaction (MFPI) analysis. Estimated treatment effect (solid cyan line) with pointwise 95% CI (shaded grey area) is shown for (A) overall and (B) failure-free survival. The horizontal line at hazard ratio 1 denotes equivalence of treatment effects. Values beneath this line indicate that prostate RT + SOC is more effective than SOC alone. The p-values shown are for treatment-aBSI interaction based on MFPI analysis.
3. **Supplementary Figure 2:** Sub-population treatment effect pattern plots (STEPP) for graphical evaluation of interaction between treatment and automated bone scan index (aBSI), constructed by varying *g* (4, 6 and 8). Upper panel: overall survival (A); lower panel: failure-free survival (B). The solid line represents the estimated treatment effects in sub-populations with 95% confidence intervals (shaded grey area).
4. **Supplementary Table 1:** Baseline cohort characteristics of patients randomized within the STAMPEDE M1|RT comparison and the patients included in this study (aBSI cohort).
5. **Supplementary Table 2:** Baseline characteristics by aBSI quartiles.
6. **Supplementary Table 3:** Baseline characteristics in aBSI quartile 1 by treatment groups.
7. **Supplementary Table 4:** Baseline characteristics in aBSI quartile 2 by treatment groups.
8. **Supplementary Table 5:** Baseline characteristics in aBSI quartile 3 by treatment groups.
9. **Supplementary Table 6:** Baseline characteristics in aBSI quartile 4 by treatment groups.
10. **Supplementary Table 7:** Hazard ratios from adjusted Cox models for outcomes within aBSI quartiles.

**Supplementary methods: Image requirements for aBSI analysis**

The aBSI was evaluated using the Exini aBSI v3.2.1 software (EXINI diagnostics, Lund, Sweden). The input required is a planar whole-body bone scans with one anterior and one posterior image acquired with a gamma camera. The anterior and posterior images should cover at least an area from the scalp to the upper part of tibia, and the upper part of antebrachium of each arm. The images may be two frames in one DICOM file or two DICOM files with a single frame each. Images must be uncompressed and in DICOM 3 format. Filtering or other post-processing techniques must not have been applied to the images. Image pixel data should be in the 16-bit range. Images with pixels ranging from 0 to 255 (8-bit) are not sufficient. The attribute criteria specified in the table below must be met.

| **Attribute** |  | **Criterion** |
| --- | --- | --- |
|  |  |  |
| **Attribute Name** | **Tag** |  |
|  |  |  |
| Image Type | 0008,0008 | ORIGINAL\PRIMARY\WHOLE BODY\EMISSION |
|  |  |  |
| SOP Class UID | 0008,0016 | Nuclear Medicine Image Storage, 1.2.840.10008.5.1.4.1.1.20 |
|  |  |  |
| Modality | 0008,0060 | NM |
|  |  |  |
| Rows | 0020,0010 | Must be >= to Columns 0028,0011 |
|  |  |  |
| Pixel Spacing | 0028,0030 | Must be >= 1.8 and <= to 2.8. Square pixels. |
|  |  |  |

**Supplementary Figure 1:** Treatment effect plots for automated bone scan index (aBSI) from a multivariable fractional polynomial interaction (MFPI) analysis. Estimated treatment effect (solid cyan line) with pointwise 95% CI (shaded grey area) is shown for (A) overall and (B) failure-free survival. The horizontal line at hazard ratio 1 denotes equivalence of treatment effects. Values beneath this line indicate that prostate RT + SOC is more effective than SOC alone. The p-values shown are for treatment-aBSI interaction based on MFPI analysis.

| **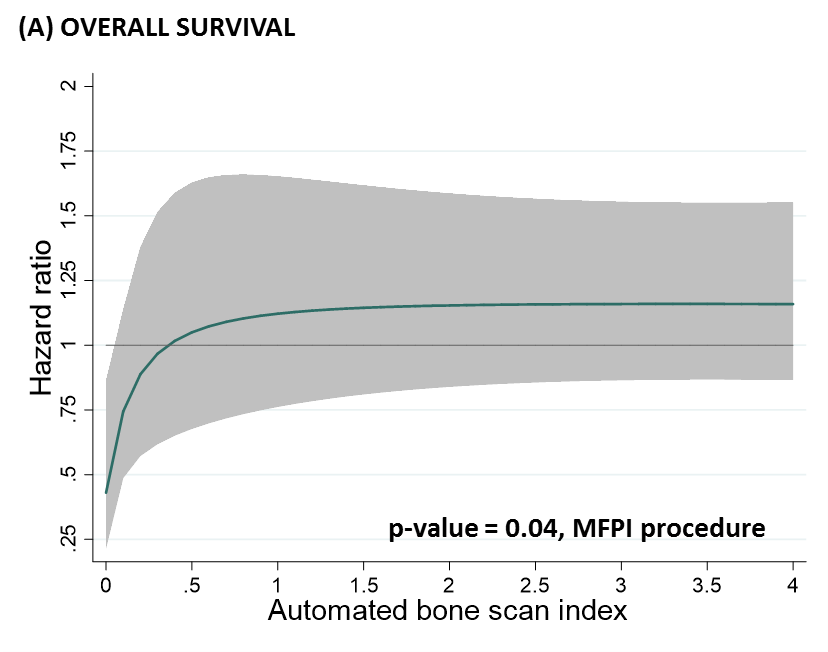** |
| --- |
| **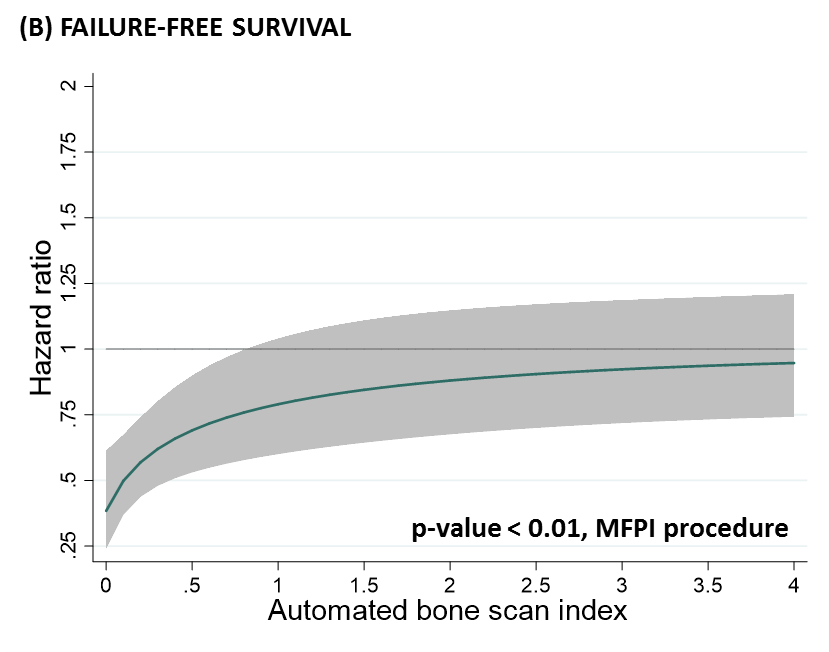** |

**Supplementary Figure 2:** Sub-population treatment effect pattern plots (STEPP) for graphical evaluation of interaction between treatment and automated bone scan index (aBSI), constructed by varying *g* (4, 6 and 8). Upper panel: overall survival (A); lower panel: failure-free survival (B). The solid line represents the estimated treatment effects in sub-populations with 95% confidence intervals (shaded grey area).

**
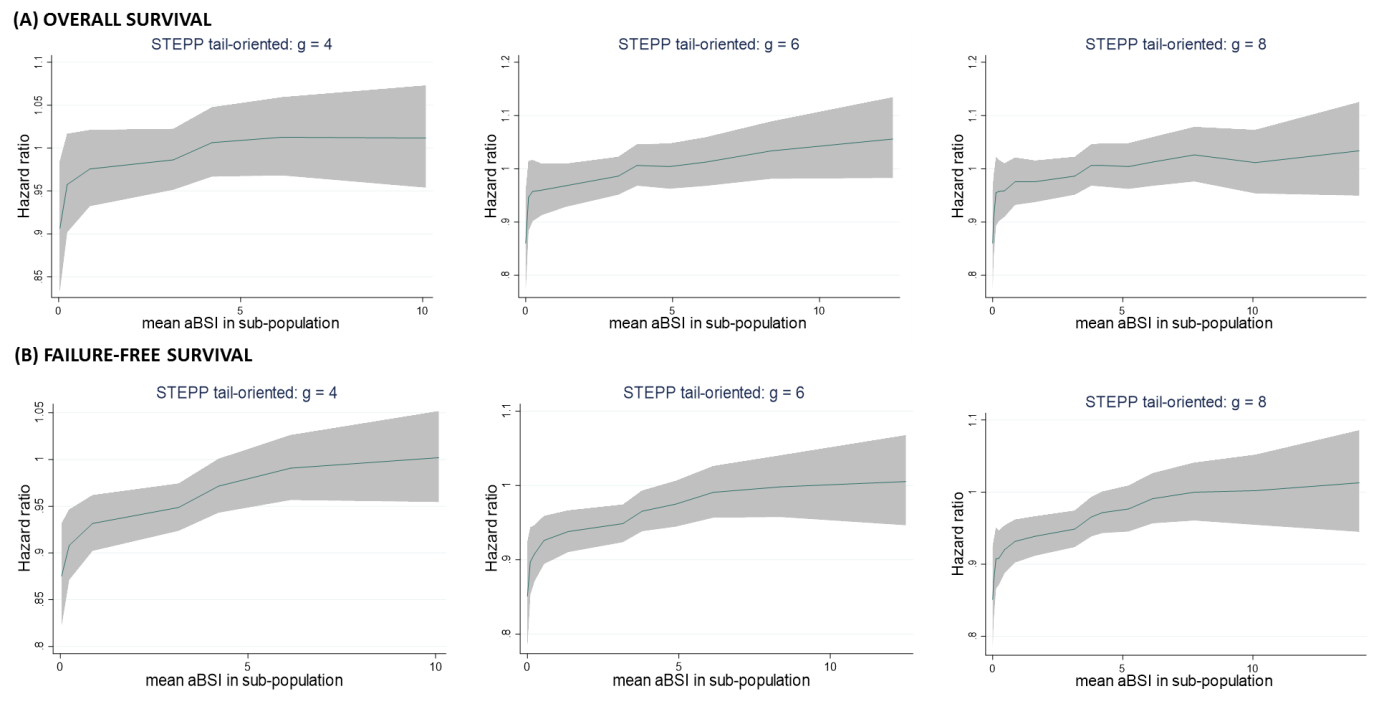
**

**Supplementary Table 1:** Baseline cohort characteristics of patients randomized within the STAMPEDE M1|RT comparison and the patients included in this study (aBSI cohort).

|  | **M1\|RT comparison (n=2061)** | | | | |  | **aBSI cohort (n=660)** | | | | |
| --- | --- | --- | --- | --- | --- | --- | --- | --- | --- | --- | --- |
|  | **SOC**  **(n=1029)** | |  | **SOC + RT (n=1032)** | |  | **SOC**  **(n=323)** | |  | **SOC + RT (n=337)** | |
|  | **n** | **%** |  | **n** | **%** |  | **n** | **%** |  | **n** | **%** |
| **Age at randomisation** |  |  |  |  |  |  |  |  |  |  |  |
| **Median** | 68 | |  | 68 | |  | 68 | |  | 68 | |
| **IQR** | 63 - 73 | |  | 63-73 | |  | 63-73 | |  | 63-73 | |
| **PSA (ng/ml) before ADT** |  |  |  |  |  |  |  |  |  |  |  |
| **Median** | 98 | |  | 97 | |  | 94 | |  | 111 | |
| **IQR** | 30 - 316 | |  | 33 - 313 | |  | 33-355 | |  | 36-331 | |
| **WHO performance status** | |  |  |  |  |  |  |  |  |  |  |
| **0** | 732 | 71 |  | 734 | 71 |  | 248 | 77 |  | 252 | 75 |
| **1 to 2** | 297 | 29 |  | 298 | 29 |  | 75 | 23 |  | 85 | 25 |
| **Primary tumour stage** |  |  |  |  |  |  |  |  |  |  |  |
| **≤T2** | 96 | 9 |  | 103 | 10 |  | 35 | 11 |  | 32 | 10 |
| **T3** | 585 | 57 |  | 603 | 58 |  | 195 | 60 |  | 205 | 61 |
| **T4** | 260 | 25 |  | 246 | 24 |  | 72 | 22 |  | 83 | 25 |
| **TX** | 88 | 9 |  | 80 | 8 |  | 21 | 7 |  | 17 | 5 |
| **Gleason score** |  |  |  |  |  |  |  |  |  |  |  |
| **≤7** | 173 | 17 |  | 172 | 17 |  | 50 | 15 |  | 64 | 19 |
| **8 to 10** | 820 | 80 |  | 810 | 78 |  | 260 | 81 |  | 261 | 78 |
| **Unknown** | 36 | 3 |  | 50 | 5 |  | 13 | 4 |  | 12 | 4 |
| **Regional node status** |  |  |  |  |  |  |  |  |  |  |  |
| **N0** | 345 | 34 |  | 344 | 33 |  | 93 | 29 |  | 111 | 33 |
| **N1** | 620 | 60 |  | 620 | 60 |  | 207 | 64 |  | 211 | 63 |
| **NX** | 64 | 6 |  | 68 | 7 |  | 23 | 7 |  | 15 | 4 |
| **Nominated RT schedule** |  |  |  |  |  |  |  |  |  |  |  |
| **36Gy in 6f over 6 weeks** | 482 | 47 |  | 497 | 48 |  | 158 | 49 |  | 179 | 53 |
| **55Gy in 20f over 4 weeks** | 547 | 53 |  | 535 | 52 |  | 165 | 51 |  | 158 | 47 |
| **Planned Docetaxel use** |  |  |  |  |  |  |  |  |  |  |  |
| **No** | 845 | 82 |  | 849 | 82 |  | 261 | 81 |  | 276 | 82 |
| **Yes** | 184 | 18 |  | 183 | 18 |  | 62 | 19 |  | 61 | 18 |
| **Metastatic sites** |  |  |  |  |  |  |  |  |  |  |  |
| **Bone** | 919 | 89 |  | 917 | 89 |  | 289 | 89 |  | 306 | 91 |
| **NRLN** | 294 | 29 |  | 304 | 29 |  | 94 | 29 |  | 95 | 28 |
| **Lung** | 42 | 4 |  | 48 | 5 |  | 10 | 3 |  | 9 | 3 |
| **Liver** | 23 | 2 |  | 19 | 2 |  | 5 | 2 |  | 3 | 1 |
| **Other** | 35 | 3 |  | 33 | 3 |  | 11 | 3 |  | 8 | 2 |
| **Abbreviations: SOC – standard of care, RT – radiotherapy, IQR – interquartile range, PSA – prostate specific antigen, ADT – androgen deprivation therapy, NRLN – non-regional lymph nodes, aBSI – automated bone scan index** | | | | | | | | | | | |

**Supplementary Table 2:** Baseline characteristics by aBSI quartiles.

|  | **aBSI quartile 1 (n=168)** | | | **aBSI quartile 2 (n=166)** | | | **aBSI quartile 3 (n=162)** | | | **aBSI quartile 4 (n=164)** | | |
| --- | --- | --- | --- | --- | --- | --- | --- | --- | --- | --- | --- | --- |
|  | **n** | | **%** | **n** | | **%** | **n** | | **%** | **n** | | **%** |
| **Age at randomisation** |  |  | |  |  | |  |  | |  |  | |
| **Median** | 68 | | | 68 | | | 69 | | | 68 | | |
| **IQR** | 63-73 | | | 63-73 | | | 63-74 | | | 62-71 | | |
| **PSA (ng/ml) before ADT** |  |  | |  |  | |  |  | |  |  | |
| **Median** | 53 | | | 64 | | | 113 | | | 479 | | |
| **IQR** | 26-123 | | | 24-146 | | | 36-331 | | | 152-1150 | | |
| **WHO performance status** |  |  | |  |  | |  |  | |  |  | |
| **0** | 132 | 79 | | 126 | 76 | | 130 | 80 | | 112 | 68 | |
| **1 to 2** | 36 | 21 | | 40 | 24 | | 32 | 20 | | 52 | 32 | |
| **Primary tumour stage** |  |  | |  |  | |  |  | |  |  | |
| **≤T2** | 16 | 10 | | 19 | 11 | | 19 | 12 | | 13 | 8 | |
| **T3** | 106 | 63 | | 108 | 65 | | 95 | 59 | | 91 | 56 | |
| **T4** | 41 | 24 | | 30 | 18 | | 40 | 25 | | 44 | 27 | |
| **TX** | 5 | 3 | | 9 | 5 | | 8 | 5 | | 16 | 10 | |
| **Gleason score** |  |  | |  |  | |  |  | |  |  | |
| **≤7** | 30 | 18 | | 40 | 24 | | 20 | 12 | | 24 | 15 | |
| **8 to 10** | 133 | 80 | | 121 | 73 | | 133 | 82 | | 134 | 82 | |
| **Unknown** | 5 | 3 | | 5 | 3 | | 9 | 6 | | 6 | 4 | |
| **Regional node status** |  |  | |  |  | |  |  | |  |  | |
| **N0** | 38 | 23 | | 61 | 37 | | 55 | 34 | | 50 | 30 | |
| **N1** | 127 | 76 | | 98 | 59 | | 100 | 62 | | 93 | 57 | |
| **NX** | 3 | 2 | | 7 | 4 | | 7 | 4 | | 21 | 13 | |
| **RT schedule** |  |  | |  |  | |  |  | |  |  | |
| **36Gy in 6f over 6 weeks** | 76 | 45 | | 82 | 49 | | 89 | 55 | | 90 | 55 | |
| **55Gy in 20f over 4 weeks** | 92 | 55 | | 84 | 51 | | 73 | 45 | | 74 | 45 | |
| **Docetaxel use** |  |  | |  |  | |  |  | |  |  | |
| **No** | 136 | 81 | | 137 | 83 | | 133 | 82 | | 131 | 80 | |
| **Yes** | 32 | 19 | | 29 | 17 | | 29 | 18 | | 33 | 20 | |
| **Metastatic sites** |  |  | |  |  | |  |  | |  |  | |
| **Bone** | 113 | 67 | | 157 | 95 | | 161 | 99 | | 164 | 100 | |
| **NRLN** | 77 | 46 | | 31 | 19 | | 39 | 24 | | 42 | 26 | |
| **Lung** | 4 | 2 | | 4 | 2 | | 5 | 3 | | 6 | 4 | |
| **Liver** | 3 | 2 | | 1 | 1 | | 1 | 1 | | 3 | 2 | |
| **Other** | 9 | 5 | | 1 | 1 | | 6 | 4 | | 3 | 2 | |
| **aBSI** |  |  | |  |  | |  |  | |  |  | |
| **Range** | 0-0.1 | | | 0.2-0.9 | | | 1-4.1 | | | 4.2-27.6 | | |
| **Number of bone metastases** |  |  | |  |  | |  |  | |  |  | |
| **≤3** | 156 | 93 | | 106 | 64 | | 23 | 14 | | 0 | 0 | |
| **4 to 6** | 10 | 6 | | 52 | 31 | | 39 | 24 | | 0 | 0 | |
| **≥7** | 2 | 1 | | 8 | 5 | | 100 | 62 | | 164 | 100 | |
| **Metastatic burden*** |  |  | |  |  | |  |  | |  |  | |
| **Low** | 151 | 90 | | 101 | 61 | | 30 | 19 | | 2 | 1 | |
| **High** | 17 | 10 | | 65 | 39 | | 132 | 81 | | 162 | 99 | |
| ***CHAARTED definition.**  **Abbreviations: SOC – standard of care, RT – radiotherapy, IQR – interquartile range, PSA – prostate specific antigen, ADT – androgen deprivation therapy, NRLN – non-regional lymph nodes, aBSI – automated bone scan index** | | | | | | | | | | | | |

**Supplementary Table 3:** Baseline characteristics in aBSI quartile 1 by treatment groups.

|  | **SOC (n=79)** | | **SOC + RT (n=89)** | |
| --- | --- | --- | --- | --- |
|  | **n** | **%** | **n** | **%** |
| **Age at randomisation** |  |  |  |  |
| **Median** | 68 | | 68 | |
| **IQR** | 63-73 | | 64-72 | |
| **PSA (ng/ml) before ADT** |  |  |  |  |
| **Median** | 48 | | 55 | |
| **IQR** | 22-112 | | 27-137 | |
| **WHO performance status** |  |  |  |  |
| **0** | 60 | 76 | 72 | 81 |
| **1 to 2** | 19 | 24 | 17 | 19 |
| **Primary tumour stage** |  |  |  |  |
| **≤T2** | 7 | 9 | 9 | 10 |
| **T3** | 50 | 63 | 56 | 63 |
| **T4** | 19 | 24 | 22 | 25 |
| **TX** | 3 | 4 | 2 | 2 |
| **Gleason score** |  |  |  |  |
| **≤7** | 10 | 13 | 20 | 22 |
| **8 to 10** | 68 | 86 | 65 | 73 |
| **Unknown** | 1 | 1 | 4 | 5 |
| **Regional node status** |  |  |  |  |
| **N0** | 17 | 22 | 21 | 24 |
| **N1** | 60 | 76 | 67 | 75 |
| **NX** | 2 | 3 | 1 | 1 |
| **Nominated RT schedule** |  |  |  |  |
| **36Gy in 6f over 6 weeks** | 39 | 49 | 37 | 42 |
| **55Gy in 20f over 4 weeks** | 40 | 51 | 52 | 58 |
| **Planned Docetaxel use** |  |  |  |  |
| **No** | 66 | 84 | 70 | 79 |
| **Yes** | 13 | 16 | 19 | 21 |
| **Metastatic sites** |  |  |  |  |
| **Bone** | 53 | 67 | 60 | 67 |
| **NRLN** | 36 | 46 | 41 | 46 |
| **Lung** | 3 | 4 | 1 | 1 |
| **Liver** | 2 | 3 | 1 | 1 |
| **Other** | 6 | 8 | 3 | 3 |
| **Metastatic burden*** |  |  |  |  |
| **Low** | 73 | 92 | 78 | 88 |
| **High** | 6 | 8 | 11 | 12 |
| **Number of bone metastases** |  |  |  |  |
| **≤ 3** | 77 | 97 | 79 | 89 |
| **4 - 6** | 2 | 3 | 8 | 9 |
| **≥ 7** | 0 | 0 | 2 | 2 |
| **aBSI** |  |  |  |  |
| **Range** | 0-0.1 | | 0-0.1 | |
| ***CHAARTED definition.**  **Abbreviations: SOC – standard of care, RT – radiotherapy, IQR – interquartile range, PSA – prostate specific antigen, ADT – androgen deprivation therapy, NRLN – non-regional lymph nodes, aBSI – automated bone scan index** | | | | |

**Supplementary Table 4:** Baseline characteristics in aBSI quartile 2 by treatment groups.

|  | **SOC (n=78)** | | **SOC + RT (n=88)** | |
| --- | --- | --- | --- | --- |
|  | **n** | **%** | **n** | **%** |
| **Age at randomisation** |  |  |  |  |
| **Median** | 69 | | 67 | |
| **IQR** | 66 - 73 | | 61 - 72 | |
| **PSA (ng/ml) before ADT** |  |  |  |  |
| **Median** | 64 | | 56 | |
| **IQR** | 22-144 | | 26-149 | |
| **WHO performance status** |  |  |  |  |
| **0** | 63 | 81 | 63 | 72 |
| **1 to 2** | 15 | 19 | 25 | 28 |
| **Primary tumour stage** |  |  |  |  |
| **≤T2** | 10 | 13 | 9 | 10 |
| **T3** | 54 | 69 | 54 | 61 |
| **T4** | 9 | 12 | 21 | 24 |
| **TX** | 5 | 6 | 4 | 5 |
| **Gleason score** |  |  |  |  |
| **≤7** | 18 | 23 | 22 | 25 |
| **8 to 10** | 57 | 73 | 64 | 73 |
| **Unknown** | 3 | 4 | 2 | 2 |
| **Regional node status** |  |  |  |  |
| **N0** | 25 | 32 | 36 | 41 |
| **N1** | 47 | 60 | 51 | 58 |
| **NX** | 6 | 8 | 1 | 1 |
| **Nominated RT schedule** |  |  |  |  |
| **36Gy in 6f over 6 weeks** | 36 | 46 | 46 | 52 |
| **55Gy in 20f over 4 weeks** | 42 | 54 | 42 | 48 |
| **Planned Docetaxel use** |  |  |  |  |
| **No** | 64 | 82 | 73 | 83 |
| **Yes** | 14 | 18 | 15 | 17 |
| **Metastatic sites** |  |  |  |  |
| **Bone** | 71 | 91 | 86 | 98 |
| **NRLN** | 17 | 22 | 14 | 16 |
| **Lung** | 3 | 4 | 1 | 1 |
| **Liver** | 0 | 1 | 1 | 1 |
| **Other** | 1 | 1 | 0 | 0 |
| **Metastatic burden*** |  |  |  |  |
| **Low** | 43 | 55 | 58 | 66 |
| **High** | 35 | 45 | 30 | 34 |
| **Number of bone metastases** |  |  |  |  |
| **≤ 3** | 47 | 60 | 59 | 67 |
| **4 - 6** | 25 | 32 | 27 | 31 |
| **≥ 7** | 6 | 8 | 2 | 2 |
| **aBSI** |  |  |  |  |
| **Range** | 0.2-0.9 | | 0.2-0.9 | |
| ***CHAARTED definition.**  **Abbreviations: SOC – standard of care, RT – radiotherapy, IQR – interquartile range, PSA – prostate specific antigen, ADT – androgen deprivation therapy, NRLN – non-regional lymph nodes, aBSI – automated bone scan index** | | | | |

**Supplementary Table 5:** Baseline characteristics in aBSI quartile 3 by treatment groups**.**

|  | **SOC (n=84)** | | **SOC + RT (n=78)** | |
| --- | --- | --- | --- | --- |
|  | **n** | **%** | **n** | **%** |
| **Age at randomisation** |  |  |  |  |
| **Median** | 70 | | 68 | |
| **IQR** | 63 - 74 | | 62 - 75 | |
| **PSA (ng/ml) before ADT** |  |  |  |  |
| **Median** | 117 | | 103 | |
| **IQR** | 31-318 | | 42-333 | |
| **WHO performance status** |  |  |  |  |
| **0** | 70 | 83 | 60 | 77 |
| **1 to 2** | 14 | 17 | 18 | 23 |
| **Primary tumour stage** |  |  |  |  |
| **≤T2** | 11 | 13 | 8 | 10 |
| **T3** | 47 | 56 | 48 | 62 |
| **T4** | 20 | 24 | 20 | 26 |
| **TX** | 6 | 7 | 2 | 3 |
| **Gleason score** |  |  |  |  |
| **≤7** | 10 | 12 | 10 | 13 |
| **8 to 10** | 69 | 82 | 64 | 82 |
| **Unknown** | 5 | 6 | 4 | 5 |
| **Regional node status** |  |  |  |  |
| **N0** | 29 | 35 | 26 | 33 |
| **N1** | 50 | 60 | 50 | 64 |
| **NX** | 5 | 6 | 2 | 3 |
| **Nominated RT schedule** |  |  |  |  |
| **36Gy in 6f over 6 weeks** | 44 | 52 | 45 | 58 |
| **55Gy in 20f over 4 weeks** | 40 | 48 | 33 | 42 |
| **Planned Docetaxel use** |  |  |  |  |
| **No** | 65 | 77 | 68 | 87 |
| **Yes** | 19 | 23 | 10 | 13 |
| **Metastatic sites** |  |  |  |  |
| **Bone** | 83 | 99 | 78 | 100 |
| **NRLN** | 20 | 24 | 19 | 24 |
| **Lung** | 2 | 2 | 3 | 4 |
| **Liver** | 1 | 1 | 0 | 0 |
| **Other** | 3 | 4 | 3 | 4 |
| **Metastatic burden*** |  |  |  |  |
| **Low** | 18 | 21 | 12 | 15 |
| **High** | 66 | 79 | 66 | 85 |
| **Number of bone metastases** |  |  |  |  |
| **≤ 3** | 16 | 19 | 7 | 9 |
| **4 - 6** | 22 | 26 | 17 | 22 |
| **≥ 7** | 46 | 55 | 54 | 69 |
| **aBSI** |  |  |  |  |
| **Range** | 1-4.1 | | 1-4 | |
| ***CHAARTED definition.**  **Abbreviations: SOC – standard of care, RT – radiotherapy, IQR – interquartile range, PSA – prostate specific antigen, ADT – androgen deprivation therapy, NRLN – non-regional lymph nodes, aBSI – automated bone scan index** | | | | |

**Supplementary Table 6:** Baseline characteristics in aBSI quartile 4 by treatment groups.

|  | **SOC (n=82)** | | **SOC + RT (n=82)** | |
| --- | --- | --- | --- | --- |
|  | **n** | **%** | **n** | **%** |
| **Age at randomisation** |  |  |  |  |
| **Median** | 66 | | 68 | |
| **IQR** | 62-71 | | 63-73 | |
| **PSA (ng/ml) before ADT** |  |  |  |  |
| **Median** | 494 | | 469 | |
| **IQR** | 128-1098 | | 162-1313 | |
| **WHO performance status** |  |  |  |  |
| **0** | 55 | 67 | 57 | 70 |
| **1 to 2** | 27 | 33 | 25 | 30 |
| **Primary tumour stage** |  |  |  |  |
| **≤T2** | 7 | 9 | 6 | 7 |
| **T3** | 44 | 54 | 47 | 57 |
| **T4** | 24 | 29 | 20 | 24 |
| **TX** | 7 | 9 | 9 | 11 |
| **Gleason score** |  |  |  |  |
| **≤7** | 12 | 15 | 12 | 15 |
| **8 to 10** | 66 | 81 | 68 | 83 |
| **Unknown** | 4 | 5 | 2 | 2 |
| **Regional node status** |  |  |  |  |
| **N0** | 22 | 27 | 28 | 34 |
| **N1** | 50 | 61 | 43 | 52 |
| **NX** | 10 | 12 | 11 | 13 |
| **Nominated RT schedule** |  |  |  |  |
| **36Gy in 6f over 6 weeks** | 39 | 48 | 51 | 62 |
| **55Gy in 20f over 4 weeks** | 43 | 52 | 31 | 38 |
| **Planned Docetaxel use** |  |  |  |  |
| **No** | 66 | 80 | 65 | 79 |
| **Yes** | 16 | 20 | 17 | 21 |
| **Metastatic sites** |  |  |  |  |
| **Bone** | 82 | 100 | 82 | 100 |
| **NRLN** | 21 | 26 | 21 | 26 |
| **Lung** | 2 | 2 | 4 | 5 |
| **Liver** | 2 | 2 | 1 | 1 |
| **Other** | 1 | 1 | 2 | 2 |
| **Metastatic burden*** |  |  |  |  |
| **Low** | 1 | 1 | 1 | 1 |
| **High** | 81 | 99 | 81 | 99 |
| **Number of bone metastases** |  |  |  |  |
| **≤ 3** | 0 | 0 | 0 | 0 |
| **4 - 6** | 0 | 0 | 0 | 0 |
| **≥ 7** | 82 | 100 | 82 | 100 |
| **aBSI** |  |  |  |  |
| **Range** | 4.2-22.9 | | 4.2-27.6 | |
| ***CHAARTED definition.**  **Abbreviations: SOC – standard of care, RT – radiotherapy, IQR – interquartile range, PSA – prostate specific antigen, ADT – androgen deprivation therapy, NRLN – non-regional lymph nodes, aBSI – automated bone scan index** | | | | |

**Supplementary Table 7:** Hazard ratios from adjusted Cox models for outcomes within aBSI quartiles.

|  |  |  | |  |
| --- | --- | --- | --- | --- |
|  | **SOC** | **SOC + RT** | **Hazard ratio (95% CI)**** | |
|  | **No. of events/no. of patients** | | |  |
| **Progression-free survival** |  |  | |  |
| aBSI Quartile 1 | 41/79 | 26/89 | | 0.43 (0.26 - 0.71) |
| aBSI Quartile 2 | 30/78 | 33/88 | | 0.91 (0.54 - 1.52) |
| aBSI Quartile 3 | 50/84 | 46/78 | | 0.92 (0.61 - 1.38) |
| aBSI Quartile 4 | 59/82 | 60/82 | | 1.09 (0.76 - 1.57) |
| **Metastatic progression-free survival** |  |  | |  |
| aBSI Quartile 1 | 37/79 | 25/89 | | 0.50 (0.30 - 0.85) |
| aBSI Quartile 2 | 26/78 | 33/88 | | 1.04 (0.61 - 1.80) |
| aBSI Quartile 3 | 46/84 | 44/78 | | 0.94 (0.62 - 1.43) |
| aBSI Quartile 4 | 59/82 | 60/82 | | 1.08 (0.75 - 1.56) |
| **Prostate cancer specific survival*** |  |  | |  |
| aBSI Quartile 1 | 23/79 | 11/89 | | 0.37 (0.17 - 0.81) |
| aBSI Quartile 2 | 13/78 | 18/88 | | 1.13 (0.53 - 2.41) |
| aBSI Quartile 3 | 25/84 | 28/78 | | 1.11 (0.64 - 1.92) |
| aBSI Quartile 4 | 43/82 | 45/82 | | 1.11 (0.72 - 1.72) |
| **Symptomatic local event-free survival** |  |  | |  |
| aBSI Quartile 1 | 34/79 | 25/89 | | 0.53 (0.31 - 0.90) |
| aBSI Quartile 2 | 23/78 | 30/88 | | 1.25 (0.71 - 2.19) |
| aBSI Quartile 3 | 37/84 | 41/78 | | 1.19 (0.76 - 1.88) |
| aBSI Quartile 4 | 44/82 | 46/82 | | 1.16 (0.76 - 1.78) |
| Abbreviations: SOC – standard of care, RT – radiotherapy, CI – confidence intervals, aBSI – automated bone scan index.  **Adjusted for age (<70 or ≥70), N stage (N0, N+ or NX), WHO PS (0 or 1-2), NSAID or aspirin use (uses either or no), planned docetaxel use (yes or no).  * Competing risk regression model adjusted for the same variable as shown above was used to evaluate sub-distribution HR for evaluating prostate cancer specific survival. | | | | |
|  |  |  | |  |
